# Early Appearance of Epicardial Adipose Tissue through Human Development

**DOI:** 10.3390/nu13092906

**Published:** 2021-08-24

**Authors:** Juliana Perez-Miguelsanz, Vanesa Jiménez-Ortega, Pilar Cano-Barquilla, Marta Garaulet, Ana I. Esquifino, Gregorio Varela-Moreiras, Pilar Fernández-Mateos

**Affiliations:** 1 Departamento de Anatomía y Embriología, Facultad de Medicina, Universidad Complutense de Madrid, 28040 Madrid, Spain; jperezm@ucm.es; 2 Instituto de Investigación Sanitaria del Hospital Clínico San Carlos (IdISSC), 28003 Madrid, Spain; jimenezv@ucm.es (V.J.-O.); mpcanoba@ucm.es (P.C.-B.); pelayos@ucm.es (A.I.E.); 3 Departamento de Bioquímica y Biología Molecular, Facultad de Medicina, Universidad Complutense de Madrid, 28040 Madrid, Spain; 4 Departamento de Fisiología, Universidad de Murcia, IMIB-Arrixaca, 30120 Murcia, Spain; garaulet@um.es; 5 Departamento de Ciencias Farmacéuticas y de la Salud, Facultad de Farmacia, Universidad CEU San Pablo, Boadilla del Monte, 28668 Madrid, Spain; gvarela@ceu.es; 6 Departamento de Biología Celular, Facultad de Medicina, Universidad Complutense, 28040 Madrid, Spain

**Keywords:** epicardial adipose tissue development, coronary arteries, human embryo and fetus, cardiovascular disease, metabolic fetal programing

## Abstract

**Background**: Epicardial adipose tissue (EAT) is a visceral fat depot with unique anatomic, biomolecular and genetic features. Due to its proximity to the coronary arteries and myocardium, dysfunctional EAT may contribute to the development and progression of cardiovascular and metabolic-related adiposity-based chronic diseases. The aim of this work was to describe, by morphological techniques, the early origin of EAT. **Methods:** EAT adipogenesis was studied in 41 embryos from 32 gestational days (GD) to 8 gestational weeks (GW) and in 23 fetuses until full term (from 9 to 36 GW). **Results:** This process comprises five stages. Stage 1 appears as mesenchyme at 33–35 GD. Stage 2 is characterized by angiogenesis at 42–45 GD. Stage 3 covers up to 34 GW with the appearance of small fibers in the extracellular matrix. Stage 4 is visible around the coronary arteries, as multilocular adipocytes in primitive fat lobules, and Stage 5 is present with unilocular adipocytes in the definitive fat lobules. EAT precursor tissue appears as early as the end of the first gestational month in the atrioventricular grooves. Unilocular adipocytes appear at the eighth gestational month. **Conclusions:** Due to its early origin, plasticity and clinical implications, factors such as maternal health and nutrition might influence EAT early development in consequence.

## 1. Introduction

The heart wall consists of an endocardial inner layer, a contractile myocardium and the outer cover, the epicardium. The epicardium is a complex of layers with a flat mesothelium connected to the myocardium by subepicardial loose connective tissue, where a large amount of white adipose tissue (AT) accumulates, forming the so-called epicardial adipose tissue (EAT) [1]. Indeed, the epicardium and the myocardium share the same microcirculation, thus having a clear functional relationship [2].

The epicardium plays an important role during cardiogenesis, providing cardiovascular cell types and instructive signals. The epicardium has emerged as a multipotent cardiovascular progenitor source with therapeutic potential for coronary smooth muscle cell, cardiac fibroblast and cardiomyocyte regeneration, owing to its fundamental role in heart development and its potential ability to initiate myocardial repair in injured adult tissues [3].

EAT, located within the pericardium, directly overlays the myocardium and coronary vessels and houses the intrinsic cardiac nervous system ganglia and plexi. EAT shares a common blood supply with the heart and is positioned to have physical, metabolic, paracrine and, possibly, neuronal effects on the heart [1].

Metabolically, EAT is considered as the visceral AT of the heart. Most EAT is white fat, but there are also small brown AT depots in perivascular regions (aorta, common carotid artery) and near the heart wall (epicardium) [4].

Increased EAT mass is known to cause local cardiac pathology as well as systemic effects, increasing the risk of metabolic syndrome. Pathologic mechanisms of EAT are thought to be mediated by vasocrine or paracrine secretions facilitated by the lack of fascia separating the EAT and the myocardium [2,5,6]. Adipose tissue development is a dynamic process. Adipocytes appear during the intrauterine period and continue to develop and expand throughout life [7]. Although adipogenesis in each stage of development follows a general pattern specific to that stage, studies have shown that adiposity in prenatal life and infancy tracks into childhood and then into adulthood [8,9,10,11,12,13].

In the adipose-based chronic disease (ABCD) context, in which obesity is addressed as an “adiposity-based chronic disease”, the incorporation of the characteristics of “adiposity” includes the total amount, distribution and function of adipose tissue. In fact, body fat mass correlates with complications embodying adverse clinical endpoints that are not always adequately reflected by the body mass index (BMI) [11]. An “adiposity-based” diagnosis allows a more specific analysis of complications determined by dysfunctional adipose tissue [14].

Several components have been shown to contribute to healthy (or unhealthy) AT growth, including genetic factors as well as prenatal and postnatal exposure to dietary, lifestyle and environmental factors. Thus, there has been an extensive effort to develop effective preventive and treatment strategies targeting these determinants of AT expansion [13].

Nowadays, imaging techniques such as echocardiography are useful to measure adult epicardial fat thickness as a new imaging biomarker [15]. These morphological measures correlate with biochemical parameters such as low-density lipoproteins (LDL) and total cholesterol levels, insulin, adiponectin and even diastolic blood pressure [16]. In this direction, some reports have shown a relationship between epicardial and perivascular fatty tissue with adipokine–cytokine levels in coronary artery disease patients [17].

Of interest, the thickness of the EAT is a predictor of fatal and nonfatal coronary events in the general population, regardless of the traditional cardiovascular risk factors [18,19,20,21]. Increased EAT volume is associated with a lengthened electrocardiogram PR interval [22], suggesting that EAT can interfere with electrical conduction through the heart, modifying the heartbeat.

In the pediatric population, the thickness of the EAT correlates with metabolic syndrome risk factors [23].

However, there is a considerable lack of information on the intrauterine development of human EAT, largely due to difficulties in accessing embryonic and fetal heart tissue, and limitations in experimental approaches applied to human studies, as rodents typically have little EAT [24,25]. Classical studies in the 1980s [26,27,28] established the first appearance of subcutaneous and visceral AT during the fetal period at the 14th week of gestation (GW, 100 mm crown-rump length, CRL) in those areas where it characteristically accumulates in the adult, but in these detailed reports, EAT, surprisingly, was not addressed.

A better understanding of the appearance and the different steps in the development of EAT in humans is required for future preventive therapeutic approaches in adulthood, obesity-associated metabolic complications and coronary artery disease, including cardiac repair and regeneration.

The aim of this work was therefore to describe the early origin of EAT on a unique population of human embryos and fetuses from the Universidad Complutense of Madrid collection, in order to provide a necessary context to address clinical issues associated with obesity, fat tissue metabolism, cardiovascular diseases and cardiac repair and regeneration in adulthood.

## 2. Materials and Methods

The basis of the present study is a part of the large collections of human embryos and fetuses from the ancient Instituto de Embriología and the Departamento de Anatomía y Embriología of the Universidad Complutense of Madrid (Spain).

Only embryos and fetuses that, after external morphological analysis and subsequent microscopic evaluation of different organs, did not present congenital malformations were included in this study. These embryos and fetuses were obtained after miscarriage and ectopic pregnancies from the Departamento de Obstetricia of the Complutense University after donation by their families to the Instituto de Embriología. Informed consent was obtained from all subjects involved in the study. The use of these specimens and the procedures followed were in accordance with the ethical standards of the Universidad Complutense of Madrid Committee on Human Experimentation (n B08/374).

We studied 41 embryos from 32 GD (5–7 mm, CS 14) to 8 GW (two months) (30 mm, CS 23) [29] at the end of the embryonic period and the onset of fetal life (Table 1), and 23 fetuses from 9 GW until full term, i.e., 36 GW (320 mm CRL), as shown in Table 2. This collection of embryos and fetuses has been studied in numerous doctoral theses and research woks on heart development and other structures (see [30,31,32], among others). From the large collection of specimens, we selected normal specimens without cardiac malformations.

Measurement of fetuses corresponds to length in millimeters (mm), and age in weeks, as previously reported [33].

All specimens were preserved in 10% formalin and subsequently prepared using standard histological procedures. Most serial sections, at 7, 8 or 10 µm, were stained with hematoxylin-eosin (HE), and a minor part was stained with Azan or Masson trichrome (Table 1 and Table 2). All procedures allowed distinguishing preadipose tissue, cell outlines and nuclei of definitive fat cells using a light microscope.

Observations and photographs were performed with a Nikon Eclipse Ci microscope with a camera with NIS Elements F imaging software (Nikon Corp., Tokyo, Japan).

Although AT develops in a continuous process, prenatal fat formation can be divided into five morphogenic phases [26,27]. For this reason, the presence or absence of AT was staged according to this histologic classification. These phases of adipogenesis were studied in cardiac grooves, where AT is found in the adult, as right and left atrioventricular grooves and as interventricular and interatrial grooves. In an attempt to standardize the points of interest as much as possible, images from the atrioventricular valves level, or the interventricular grooves with the anterior descendent artery in the apex, were considered.

## 3. Results

During human development, the heart undergoes very rapid changes. It appears very early in the development, at 17–19 gestational days (GD) (Carnegie stage, CS 8), starts to beat at 21 GD and completes morphogenesis at 56–60 GD (CS 23), in accordance with the end of the embryonic period, but it continues growth until delivery [34]. The morphological differentiation of EAT at the microscopic level is first observed in the atrioventricular grooves of the embryonic hearts at the fifth week of gestation. It is in these grooves where fat accumulates in a greater quantity in the adult heart.

**Figure 1:**
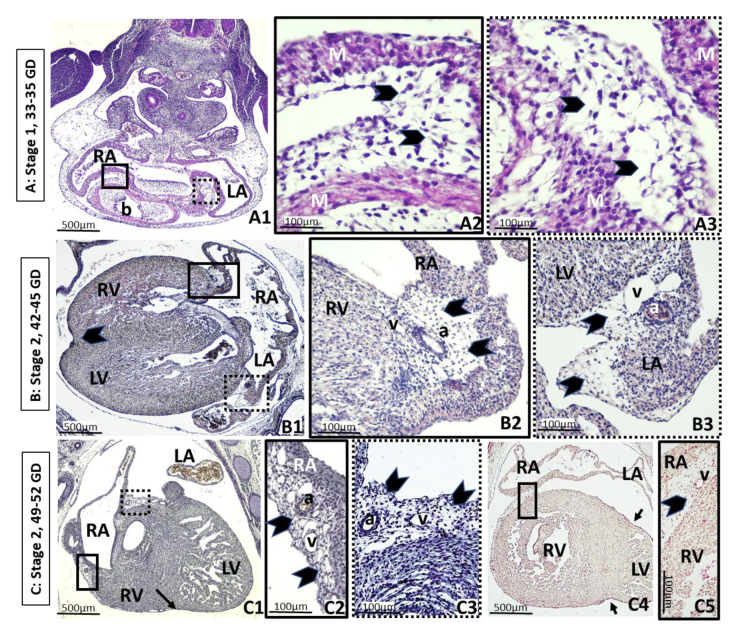
Embryonic period. a: Coronary artery. b: Cardiac bulbus. CS: Carnegie stage. GD: Gestational day. M: Myocardium. LA: Left atrium. LV: Left ventricle. RA: Right atrium. RV: Right ventricle. v: Coronary vein. A: Stage 1. Axial oblique sections of a 33–35 GD embryo. (**A1**): General view. The back is on the top of the image, and the developing heart is at the lower part. The square of solid lines delimits the right auricle-bulbus groove. The square of dotted lines marks the left auricle-bulbus groove. Successive higher-power magnification of rectangles showing loose undifferentiated mesenchyme tissue (arrowheads) composed of an amorphous ground substance of fibers and cells with a spindle-like morphology, corresponding to stage 1 of adipose tissue (AT) development, in the right (**A2**) and left (**A3**) auricle-bulbus grooves. B: Stage 2. Axial section of a 42–45 GD embryo. (**B1**): General view of the heart, which is analogous to the adult. Atrioventricular (squares) and interventricular (arrow) grooves are clearly identifiable. The interventricular groove shows vessels, but no mesenchymal tissue. (**B2**): Higher-power magnification of the square of solid lines in (**B1**), which marks the right atrioventricular groove. (**B3**): Higher-power magnification of square of dotted lines in (**B1**), which marks left atrioventricular groove. In both places, mesenchymal cells differentiate into stellate preadipocytes (arrowheads) around the coronary vessels. The coexistence of several stages of development can be observed. C: Stage 2. Sagittal section of a 49–52 GD embryo, the end of the embryonic period. (**C1**): General view of the heart. Mesenchymal tissue is placed in the atrioventricular grooves (squares) and right ventricle, but not in the interventricular groove (arrow). (**C2**): Higher-power magnification of the square of solid lines in (**C1**), which marks the right atrioventricular groove. (**C3**): Higher-power magnification of square of dotted lines in (**C1**), which marks left atrioventricular groove. In both places, condensation of the mesenchyme (arrowheads) continues as well as vascular proliferation. (**C4**): General view of a section showing both ventricles. The interventricular grooves (arrows) do not have mesenchymal cells. The right ventricle is partially covered by a mesenchymal layer similar to the atrioventricular grooves (square). (**C5**): Higher-power magnification of the square in (**C4**), showing the veins and the mesenchyme condensation (arrowhead). The coexistence of several stages of development is observed.

Stage 1 of future EAT consists of loose undifferentiated mesenchymal tissue composed of an amorphous ground substance, fibers and cells with a spindle-like morphology, as seen in embryos at 33–35 GD (5–7 mm, CS14) (Figure 1(A1–A3)). In this early heart, the undifferentiated mesenchyme layer is visible on the back and superior portion of the cardiac bulbus, as well as in the bulbus-ventricular sulci and the future left and right atrioventricular grooves. This layer is absent in the most caudal part of the primitive ventricle. At this time, there is a unique auricle because the interatrial septum has not yet formed, and the posterior wall of the atrium is attached to the chest wall, where the interatrial groove will be located. In addition, the coronary vessels are not formed at this early age (Figure 1(A1–A3)).

Stage 2 starts around one week later, being characterized by proliferation of primitive vessels or angiogenesis associated with mesenchymal condensation, which is clearly visible in embryos of 42–45 GD (13–17 mm length, CS 18) (Figure 1(B1–B3)). At that age of development, the heart presents enormous changes, septa are developed and the ventricles and atria are easily recognizable. At this moment, coronary arteries develop with a rich capillary venous network around them, lying in the atrioventricular and interventricular grooves. In stage 2 of EAT development, mesenchymal cells differentiate into stellate preadipocytes, which do not contain lipid droplets yet. Abundant stellate preadipocytes are placed in the interatrial groove as well as in the right and left atrioventricular grooves surrounding early coronary vessels but lack in the interventricular groove where the interventricular anterior vessels flow alone between the epicardium and myocardium layers (Figure 1(B1–B3)).

**Figure 2:**
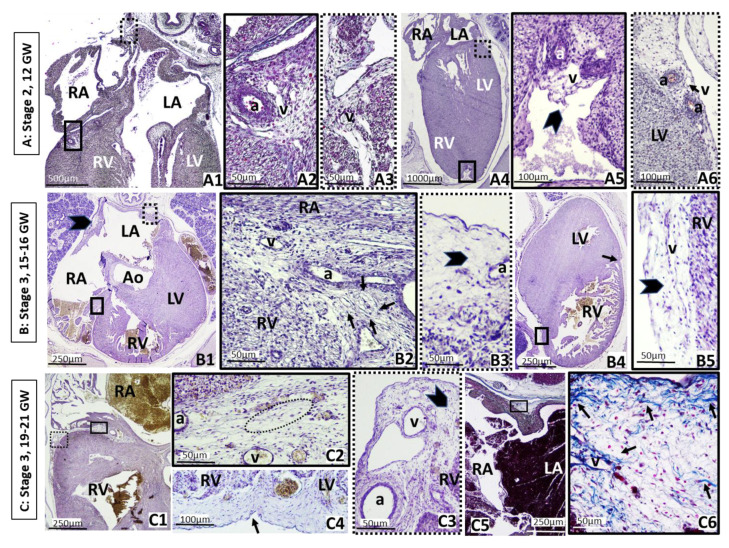
Fetal period. Coexistence of stages 1, 2 and 3. a: Coronary artery. Ao: Aorta artery. GW: Gestational week. LA: Left atrium. LV: Left ventricle. RA: Right atrium. RV: Right ventricle. v: Coronary vein. A: Stage 2. Axial oblique sections of a 12 GW fetus, the end of the first trimester. (**A1**): General view of the heart. The condensation of mesenchyme continues, with vascular proliferation in the atrioventricular grooves. A new accumulation of mesenchymal cells is visible on the posterior wall of the left auricle, next to the interatrial groove. (**A2**): Higher-power magnification of the square of solid lines in (**A1**), which marks the right atrioventricular groove with mesenchymal cells surrounding the right coronary vessels. (**A3**): Higher-power magnification of the square of dotted lines in (**A1**), showing the posterior wall of the left auricle, where vessels are developing. (**A4**): Axial oblique section from a different fetus of 12 GW. General view of the heart. (**A5**): Higher-power magnification of the square of solid lines in (**A4**), showing the onset of mesenchyme accumulation in the interventricular groove (arrow). (**A6**): Higher-power magnification of the square of dotted lines in (**A4**), showing the condensed mesenchymal cells typical of stage 2. B: Stage 3. Axial sections of 15–16 GW fetus, second trimester. (**B1**): General view of the heart. The accumulation of mesenchyme continues in the atrioventricular grooves and the posterior wall of auricles (arrowhead). (**B2**): Higher-power magnification of the square of solid lines in (**B1**), where the cells are organized in primitive mesenchymal lobes (arrows) next to the right coronary artery. (**B3**): High-power magnification of the square of dotted lines in (**B1**), showing the mesenchymal cells on the wall of the left atrium. (**B4**): Section through the ventricles, showing mesenchymal cells on the anterior (arrow) and the posterior interventricular sulcus (square). (**B5**): Higher-power magnification of the square in (**B4**), showing the mesenchymal cells on the wall of the right ventricle next to the vasculature. 3: Stage C. Axial sections of a 19–21 GW fetus, second trimester. (**C1**): General view of the right atrium and ventricle. The accumulation of mesenchyme continues in the atrioventricular grooves and the posterior wall of the ventricle. (**C2**): Higher-power magnification of the square of solid lines in (**A1**), which marks the right atrioventricular groove with primitive mesenchymal lobes surrounding the right coronary vessels (arrows) corresponding to stages 2 and 3. (**C3**): High-power magnification of the square of dotted lines, showing well-developed vasculature immersed in the mesenchyme on the posterior right ventricular wall. (**C4**): Accumulation of epicardial mesenchyme on the interventricular groove (arrow). (**C5**): General view of the posterior interatrial sulcus. (**C6**): Higher-power magnification of the square in (**C5**). Arrows show connective tissue partitions or septa next to the vasculature.

Near the end of the embryonic period, at 49–52 GD (24 mm length, CS 21), the condensation of the mesenchyme continues from stage 2, coinciding with the vascular proliferation, still limited to the atrioventricular grooves, although it begins to spread over the adjacent auricles and right ventricle. Most of the surface of the atria and ventricles is covered by a monolayer of the epicardium (Figure 1(C1–C5) and Figure 2(A1–A2)).

Stage 3 is characterized by the mesenchymal cells proliferating into stellate preadipocytes within a vascular matrix, where small fibers appear in the extracellular matrix of the condensed mesenchymal tissue, the beginning of fibrous septa, as seen in fetuses of 15–16 GW. At this stage, the organization of mesenchymal lobules begins, which contain stellate cells or preadipocytes, within a vascular structure or glomerulus, and that ultimately form a definitive fat lobule, although they still do not contain lipid droplets. These stages would cover the end of the first, the second and the beginning of the third trimester of pregnancy, up to the end of pregnancy approximately. In that time, the mesenchymal tissue proliferates considerably, accumulating in the right and left atrioventricular grooves and adjacent areas, being distributed along the right ventricle, the posterior interatrial sulcus and the dorsal part of the fetal auricle, enveloping a large part of the atria. It is thickest at the base of the heart along the lateral wall of the ventricle and thins from this point toward the ventricular septum and the base of the heart and the ventricles in the area adjacent to the grooves, leaving the cardiac apex still uncovered (Figure 2(B1–B5), (C1–C6)).

**Figure 3:**
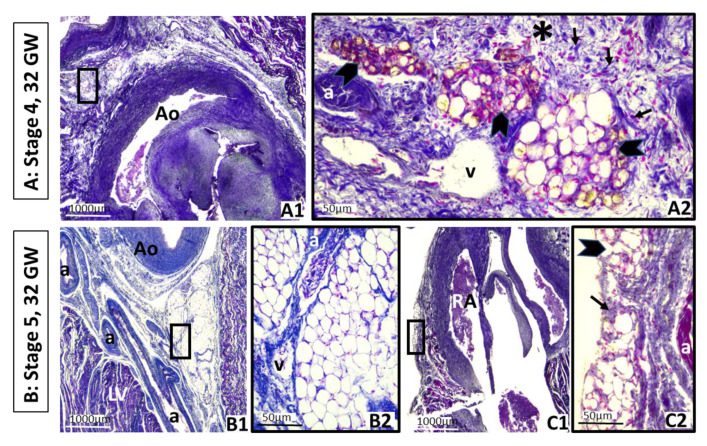
Final fetus period. Heart has grown considerably. Axial oblique sections of a 32–33 GW fetus, third trimester. Coexistence of stages 1, 2, 3, 4 and 5. a: Coronary artery. Ao: Aorta artery. GW: Gestational week. LV: Left ventricle. RA: Right atrium. v: Coronary vein. **A**: Stage 4. (**A1**): Left atrioventricular groove. Primitive fat lobes around the aorta artery are clearly visible. (**A2**): Higher-power magnification of the square in (**A1**), which marks the primitive fat lobes with fat vacuoles in cells (arrowheads) and connective tissue partitions or septa next to the left coronary vessels (black arrows), intermingled with the minor differentiated and condensed mesenchyme (asterisk). It cannot be excluded that some of the developing perivascular adipose tissue may correspond to brown fat. Coexistence of stages 1, 2 and 3. **B**: Stage 5. (**B1**): Left coronary artery on the left atrioventricular groove, next to its origin in the aorta. (**B2**): Higher-power magnification of the square in (**B1**), showing definitive fat lobes of fat cells. **C:** Stage 5. (**C1**): Epicardium on the right atrioventricular groove and posterior wall of the right atrium. (**C2**): Higher-power magnification of the square in (**C1**), showing definitive fat lobes, with fat cells (black arrowheads) and connective septa (black arrows) in this region.

At stage 4, the primitive fat lobes appear. These are visible around the coronary arteries, as seen in a fetus of 32 GW (290 mm LCR) (Figure 3(A1,A2)). Within the primitive mesenchymal lobules, fine fat vacuoles form in the cytoplasm of mesenchymal cells that progressively increase in size. Primitive fat lobules include a vascular network or glomerulus as well as densely packed vacuolated fat cells, adjacent to the small coronary vessels. It cannot be excluded that some of the developing perivascular AT may correspond to brown fat. The well-developed and primitive fat lobes are placed very close to the branching of the coronary arteries from the aorta artery. At this time, the cardiac apex is already covered by mesenchymal tissue containing stellate cells. As we have already mentioned, the human samples used belong to a collection that does not allow additional studies that differentiate brown from white fat in the early stages, except for visual identification.

In any case, the presence of fat (white or brown) has not been previously described in these very early stages.

Stage 5 represents the appearance of definitive fat lobes with mature adipocytes, well separated from each other by dense septa of perilobular mesenchymal tissue, also visible at 32 GW (Figure 3(B1–C4)). At this time, we can find fat lobes in the atrioventricular as well as in the interventricular sulcus, nearest to the coronary vessels. Note that in the sulci, connective tissue still undifferentiated surrounds the fat lobes. At that time, stages 2, 3, 4 and 5 overlap in the atrioventricular sulci since the development of EAT is continuous and the stages overlap, not being exclusive. Definitive fat lobes also appear on the atrial wall.

This study also shows that EAT formation starts in the atrioventricular grooves and progresses to the interatrial groove and right ventricle, finally reaching the interventricular groove.

**Figure 4:**
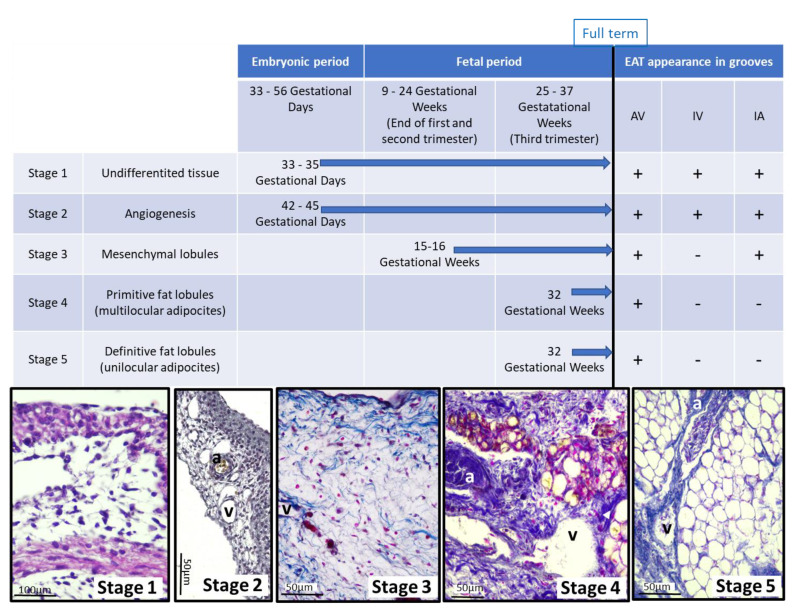
Summary of EAT appearance in human development. AV: Atrioventricular grooves; IV: interventricular groove; IA: interarticular groove. a: Coronary artery. v: Coronary vein. Presence (+); absence (-).

In summary, our data show, for the first time, that EAT appears in the embryonic period. Stage 1 of future EAT development appears as loose undifferentiated mesenchyme tissue at days 33–35 of gestation. Stage 2 is present at 42–45 GD, characterized by angiogenesis. Stage 3 covers the end of the first, the second and the beginning of the third trimester of pregnancy, up to week 34 approximately, with the appearance of small fibers in the extracellular matrix which are to be organized into lobes. Stage 4 is visible around the coronary arteries in a fetus of 32 GW, which shows multilocular adipocytes in the primitive fat lobules. At 32 GW, stage 5 is also present with unilocular adipocytes in the definitive fat lobules during EAT development, whereas stages 1 to 5 overlap in the atrioventricular groove (Figure 4).

## 4. Discussion

In the present work, we demonstrate, for the first time, that precursor tissue of EAT appears in the embryonic period as early as the beginning of the second gestational month (days 33–35 of gestation), and unilocular adipocytes are present at the eighth gestational month (week 32 of gestation). We also show that EAT formation starts in the atrioventricular grooves and progresses to the interatrial groove and right ventricle, finally reaching the interventricular groove. This is the first study about human EAT in these early stages of development until delivery.

Our results show that, as early as 33–35 GD (5–7 mm, CS 14), mesenchymal cells appear in the areas where EAT will be located in the adult, an earlier time than previously thought [26,27,28]. It is generally thought that this occurs during the last trimester of pregnancy when AT appears in humans, and when the majority of AT is deposited [26,27]. However, our data show that in this period, mature adipocytes would already be present.

Previous classical studies [26,27,28] have shown that subcutaneous AT in human fetuses starts in the head and neck at 14 GW and rapidly progresses to the trunk and thereafter to the limbs and established that adipogenesis is not a homogeneous process and each AT deposit has its own rate. However, we demonstrate that EAT develops at different times in distinct regions of the heart. In this way, the appearance in the atrioventricular grooves and the asymmetric distribution of EAT in the ventricles are in concordance with a complex structure with spatial differences aligned to the developing chambers, with distinct properties between the ventricular and the atrial epicardium [6].

Our results also show that in fetuses of 32 weeks of gestation, lobules exhibit a depot-specific architecture with marked fibrous septa, and a morphology similar to that described previously for visceral fat [35]. In this line, EAT behaves similarly to other adipose tissues such as subcutaneous and visceral AT, in which the progenitor cell subset repartition is different. Lobules exhibit a depot-specific architecture with an intrinsic capacity to remodel based on different niches of progenitor cell subsets. Those differences contribute to the intrinsic capacity of healthy/unhealthy expansion according to the fat depot [36]. At this stage, it cannot be excluded that some of the developing coronary AT may correspond to brown fat, suggesting that it may contribute to heat production at birth [4].

The role of EAT in humans is diverse. In the state of high energy demand, EAT fuels the heart with free fatty acids, the primary metabolic source of the contracting myocardium. In the same way, it prevents cardiac lipotoxicity, is a thermoregulator, offers an additional layer of mechanical protection, cushioning the heart, protects the coronary arteries against the torsion of arterial pulse waves and cardiac contraction and provides immunological support [37]. These important functions could justify its early origin.

A major limitation in understanding the biology of epicardial fat, i.e., visceral fat directly abutting the myocardium, is its reported absence in commonly used experimental animal models such as laboratory mice and rats [38]. Although mice do not have epicardial fat per se (i.e., no direct contact with the myocardium except for attachment at the atrial-ventricular groove) [39], they do have a fat depot around the heart, for which scientists refer to as pericardial fat.

The EAT of the AV sulcus in mice appears between the first and second postnatal weeks. The conditions in the AV groove of postnatal mice could favor continued mesenchymal transformation and in a manner that allows the expression and activation of peroxisome proliferator-activated receptor γ (PPARγ), which could explain the postnatal formation of EAT in this location [39]. These same authors suggested that human ventricular epicardial cells express PPARγ in a manner that would result in the presence of EAT throughout the ventricle in human neonates and subsequently throughout adult life. However, we demonstrate the presence of adipocytes before birth at 32 GW.

Due to its early origin, plasticity and clinical implications, not only the morphology and development of EAT needs to be assessed but also factors such as maternal health and nutrition, which could influence its proper development.

AT development is regulated by complex interactions of maternal, endocrine and paracrine influences that initiate specific changes in angiogenesis, adipogenesis and metabolism [40]. In the epigenetic literature, there is evidence that the entire embryo-fetal and perinatal period of development plays a key role in the early programming of all human organs and tissues. Therefore, the molecular mechanisms involved in the epigenetic programming require a new and general pathogenic paradigm, the Developmental Origins of Health and Disease theory, to explain the current epidemiological transition, that is, the worldwide increase in chronic, degenerative and inflammatory diseases such as obesity, diabetes, cardiovascular diseases, neurodegenerative diseases and cancer (see [41,42] for review). This raises the question of whether special attention should be directed to pregnant women from 33–35 GD or 5 GW (beginning of the second month of gestation).

Prenatal maternal health and nutritional status have been shown to contribute to obesity development during childhood and adolescence [43,44].

Epidemiological studies report that the timing of maternal nutrient restriction has a major influence on outcome in terms of predisposing the resulting offspring to adult obesity [45]. Nevertheless, there are some discussions about this point because some authors do not identify consistent associations between maternal diet and measures of fetal growth and adiposity in overweight and obese women [46]. More recently, another report described those fetuses from gestational diabetes mellitus pregnancies have significantly higher fetal and maternal EAT thickness values compared to non-gestational diabetes mellitus pregnancies [47,48,49] and are predisposed to developing obesity during childhood, generally between the ages of 5 and 9 years. On the other hand, maternal diet quality (assessed by the Healthy Eating Index-2015) during pregnancy and lactation was positively associated with infant percent body fat (%BF) and fat mass (in kg, by air displacement plethysmography, ADP) at 6 months of age [50]. Specifically, VAT thickness (measured by ultrasound) in the first trimester explained the variations in the newborn birth weight centile to the greatest extent compared to subcutaneous adipose tissue and BMI [51].

In this same direction, very early embryo echography, from 8 weeks of gestation [49], can be used to assess cardiac structures and adiposity. Our data show that this new knowledge is relevant to promoting the monitoring of prenatal EAT in the early stages as soon as the technique allows, in order to help the design of preventive therapeutic approaches during pregnancy. EAT thickness can be measured by echocardiography during pregnancy. This measurement has been proven to be both accurate and reproducible [52]. Recent advances in ultrasound techniques would allow the analysis of EAT to be included as a new marker in the current protocol for the morphological identification of possible cardiac pathologies related to obesity and metabolic diseases [46,47].

In view of our results, precursor tissue of EAT appears in the embryonic period as early as the beginning of the second gestational month (days 33–35 of gestation), and unilocular adipocytes are present at the eighth gestational month (week 32 of gestation). Since the thickness of EAT correlates with anthropometric and metabolic parameters in obese children with risk factors for metabolic syndrome, it would be advisable to insist on the importance of maternal health and nutrition from the beginning of pregnancy, as well as ultrasound monitoring of the heart to assess normal EAT development in the grooves of the heart, where the coronary arteries are forming, as a complement to the diagnosis of major heart defects [49,53].

## 5. Strengths and Limitations of This Study

The strengths of this study include the following:

The development of epicardial fat was studied at all stages of human heart development.

In the present work, we demonstrate, for the first time, that precursor tissue of EAT appears in the embryonic period as early as the beginning of the second gestational month (days 33–35 of gestation), and definitive lobules with unilocular adipocytes are present at the eighth gestational month (week 32 of gestation).

We also show that EAT formation starts in the atrioventricular grooves and progresses to the interatrial groove and right ventricle, finally reaching the interventricular groove. This is the first study about human EAT in these early stages of development until delivery. This suggests that special attention should be directed to pregnant woman from the 33rd–35th gestational days or the 5th gestational week (beginning of the second month of gestation). We believe that it is especially important that the morphology images are published and highlighted, since they also show that the epicardial adipose tissue begins to develop from 33–35 GW, and definitive lobules with the presence of unilocular adipocytes are observed at 32 GW, and this is something that has never been described before.

In any case, classical morphology cannot be disregarded because, in many cases, these studies are the basis for more complex molecular work [54].

The limitations of this study include the following:

This is a descriptive study without the possibility of molecular studies. The number of specimens is not very high, but the specimens include the entire gestational period.

## 6. Conclusions

Our results show that the process of epicardial adipogenesis begins at a very early stage of heart development during embryogenesis. Special attention should be directed to pregnant women from 33–35 GD or 5 GW (beginning of the second month of gestation). The results highlight the importance of monitoring the development of prenatal EAT at the early stages (week 8 of gestation if echocardiography allows) as a marker of possible adiposity-based chronic diseases in the future.

A better understanding of the mechanisms regulating the development of EAT is required for future preventive therapeutic approaches in adulthood and obesity-associated metabolic complications, including cardiac repair and regeneration.

## Figures and Tables

**Table 1:** Human embryos analyzed and characteristics.

Age (Gestational Days)	Length (mm)	Carnegie Stages (CS)	*n*	Name of Embryo	Section Orientation	Section Thickness (µm)	Stain
33–35	5 to 7	14	3	MS-5	Axial/Oblique	7	HE
				JD-2	Axial/Oblique	10	HE
				MAT-24	Axial/Oblique	10	HE
35–37	7 to 9	15	3	PV-8	Transverse	7	HE
				GI-7	Transverse	10	HE
				GV-4	Transverse	8	HE
37–40	9 to 11	16	6	MS	Transverse	8	HE/Trichrome
				DD-10	Transverse	8	HE
				FE-1	Transverse	10	HE/Trichrome
				BI-12	Transverse	7	HE
				NG-2	Transverse	7	HE/Trichrome
				mar-03	Frontal	10	HE/Trichrome
39–42	11 to 14	17	4	VE-4	Transverse	10	HE
				A-3	Transverse	7	HE
				F-23	Axial/Oblique	7	HE/Trichrome
				ES-14	Transverse	7	HE
42–45	13–17	18	5	NO	Transverse	10	HE
				FO	Transverse	7	HE/Trichrome
				C-7	Transverse	7	HE/Trichrome
				ES-15	Transverse	10-nov	HE
				MARC-1	Transverse	15–16	HE/Trichrome
45–47	17–20	19	7	PA	Transverse	10	HE
				ES-18	Transverse	7	HE/Trichrome
				ES-19	Sagittal	07-ago	HE/Trichrome
				C-9	Transverse	8	HE/Trichrome
				MM-20	Sagittal	8	HE
				ES-20	Transverse	8	HE
47–50	21–23	20	4	ES-22	Transverse	10	HE
				BI-22	Transverse	7	HE/Trichrome
				F27	Transverse	7	HE/Trichrome
				AC-23	Transverse	7	HE/Trichrome
49–52	22–24	21	2	GV-7	Sagittal	10	HE
				HA-24	Sagittal	8	HE/Trichrome
52–55	25–27	22	4	F-8	Transverse	10	HE
				MAL-25	Sagittal	8	HE
				JP-25	Transverse	8	HE
				C-27	Sagittal	8	HE/Trichrome
50–63	28–31	23	3	C11	Transverse	10	HE/Trichrome
				Mes-2	Transverse	10	HE/Trichrome
				CA-4	Transverse	10	HE
Total			41				

HE: hematoxylin-eosin.

**Table 2:** Human fetuses analyzed and characteristics.

Proposed Age (Gestational Weeks)	Crown–Rump Length (mm)	Name of the Fetus	Section Orientation	Section Thickness (µm)	Stain
9 to 11	38	OY	Sagittal	10	HE
	39–40	Faus-2	Transverse	10	HE/Trichrome
	45	F45	Sagittal	8	HE
	45	F1	Transverse	9	HE
	46	F46	Transverse	8	HE
	47	BE	Sagittal	10	HE/Trichrome
	56	Mall-28	Transverse	7	HE
	57	MA57	Transverse	7	HE
	60	F25	Sagittal	7	HE/Trichrome
	60	VS	Transverse	10	HE
	64	X	Transverse	10	HE/Trichrome
12 to 15	74.5	VR74	Transverse	10	HE/Trichrome
	79	F79	Sagittal	8	HE
	88	F88	Transverse	7	HE
	99	F99	Transverse	7	HE
	107	NO-6	Transverse	10	HE/Trichrome
	113	B-62	Frontal	10	HE/Trichrome
	117	B-29	Frontal	10	HE/Trichrome
	119	Bu-119	Transverse	10	HE/Trichrome
16–18	127	ES127	Transverse	7	HE
	137	Cu-2	Transverse	10	HE/Trichrome
32	320	EA32	Transverse	7	HE/Trichrome
Full term	305	A	Transverse	7	HE/Trichrome
Total		23			

HE: hematoxylin-eosin.

## Data Availability

The embryos and fetuses of the present work are on deposit at the Departamento de Anatomía y Embriología of the Facultad de Medicina of the Universidad Complutense of Madrid (Spain) and can be consulted on request at the head of the department.
